# Pax7 as molecular switch regulating early and advanced stages of myogenic mouse ESC differentiation in teratomas

**DOI:** 10.1186/s13287-020-01742-3

**Published:** 2020-06-17

**Authors:** Anita Florkowska, Igor Meszka, Magdalena Zawada, Diana Legutko, Tomasz J. Proszynski, Katarzyna Janczyk-Ilach, Wladyslawa Streminska, Maria A. Ciemerych, Iwona Grabowska

**Affiliations:** 1 Department of Cytology, Institute of Developmental Biology and Biomedical Sciences, Faculty of Biology, University of Warsaw, Warsaw, Poland; 2 Laboratory of Neurobiology, Nencki Institute of Experimental Biology, Polish Academy of Sciences, Warsaw, Poland; 3 Laboratory of Synaptogenesis, Nencki Institute of Experimental Biology, Polish Academy of Sciences, Warsaw, Poland; 4 Present Address: Lukasiewicz Research Network - PORT Polish Center for Technology Development, Wroclaw, Poland

**Keywords:** Mouse, embryonic stem cell, Differentiation, Teratoma, Pax7, Myogenesis, Neuromuscular junction

## Abstract

**Background:**

Pluripotent stem cells present the ability to self-renew and undergo differentiation into any cell type building an organism. Importantly, a lot of evidence on embryonic stem cell (ESC) differentiation comes from in vitro studies. However, ESCs cultured in vitro do not necessarily behave as cells differentiating in vivo. For this reason, we used teratomas to study early and advanced stages of in vivo ESC myogenic differentiation and the role of Pax7 in this process. Pax7 transcription factor plays a crucial role in the formation and differentiation of skeletal muscle precursor cells during embryonic development. It controls the expression of other myogenic regulators and also acts as an anti-apoptotic factor. It is also involved in the formation and maintenance of satellite cell population.

**Methods:**

In vivo approach we used involved generation and analysis of pluripotent stem cell-derived teratomas. Such model allows to analyze early and also terminal stages of tissue differentiation, for example, terminal stages of myogenesis, including the formation of innervated and vascularized mature myofibers.

**Results:**

We determined how the lack of Pax7 function affects the generation of different myofiber types. In Pax7−/− teratomas, the skeletal muscle tissue occupied significantly smaller area, as compared to Pax7+/+ ones. The proportion of myofibers expressing Myh3 and Myh2b did not differ between Pax7+/+ and Pax7−/− teratomas. However, the area of Myh7 and Myh2a myofibers was significantly lower in Pax7−/− ones. Molecular characteristic of skeletal muscles revealed that the levels of mRNAs coding Myh isoforms were significantly lower in Pax7−/− teratomas. The level of mRNAs encoding Pax3 was significantly higher, while the expression of *Nfix*, *Eno3*, *Mck*, *Mef2a*, and *Itga7* was significantly lower in Pax7−/− teratomas, as compared to Pax7+/+ ones. We proved that the number of satellite cells in Pax7−/− teratomas was significantly reduced. Finally, analysis of neuromuscular junction localization in samples prepared with the iDISCO method confirmed that the organization of neuromuscular junctions in Pax7−/− teratomas was impaired.

**Conclusions:**

Pax7−/− ESCs differentiate in vivo to embryonic myoblasts more readily than Pax7+/+ cells. In the absence of functional Pax7, initiation of myogenic differentiation is facilitated, and as a result, the expression of mesoderm embryonic myoblast markers is upregulated. However, in the absence of functional Pax7 neuromuscular junctions, formation is abnormal, what results in lower differentiation potential of Pax7−/− ESCs during advanced stages of myogenesis.

## Background

Pluripotent stem cells (PSCs), such as embryonic stem cells (ESCs), serve as a tool to analyze the processes responsible for the cellular differentiation occurring during mammalian development. Under appropriate culture conditions, ESCs self-renew and sustain their undifferentiated character but at the same time they are ready to undergo differentiation into any cell type building an organism [1]. Unfortunately, despite many years of research, some aspects of molecular mechanisms driving the differentiation of PSCs into functional myofibers remain obscure. In vitro cultured mouse ESCs are unable to differentiate spontaneously into fully formed, i.e., innervated and vascularized, skeletal muscle tissue. The most common in vitro approach to induce myogenic differentiation of ESCs depends either on the formation of embryoid bodies (EBs) and EB outgrowths [2–7] or ESC incubation in the presence of demethylating agents [3, 8]. Unfortunately, these methods are rather inefficient and poorly controllable. Myogenic differentiation could be enhanced by ESC genetic modifications or specific culture conditions involving complicated culture schemes recapitulating environmental changes occurring during embryonic myogenesis ([8–11], reviewed in [12, 13]). However, even under such conditions, differentiation of ESCs is limited to the early stages of myogenesis.

One of the in vivo experimental models allowing ESCs to differentiate into myofibers is based on the generation of non-malignant tumors, so-called teratomas. This unfortunately underestimated model bases on the fact that ESCs injected under mouse skin or into intracapsular space of the kidney form teratomas composed of variety of cells and tissues originating from all three germ layers [14]. The teratoma assay enables investigation of ESC spontaneous differentiation in vivo. Importantly, it also includes signals resulting from three-dimensional cell-to-cell interactions. Many studies validated such in vivo model as suitable for studying the differentiation of PSCs of various species, including mouse [15, 16] and human [17–19]. For example, teratomas obtained from induced pluripotent stem cells (iPSCs) derived from fibroblasts of Ts65Dn mouse mutant were used to follow the mechanism of reduced cancer incidence observed in humans with Down’s syndrome. As a result of this study, significantly compromised blood vessel network was determined as responsible for reduced tumor growth rather than, as suggested in previous research, anticancer effect of genes located in chromosome 21 [20]. Such model was also successfully applied to study the expression of genes coding muscle-related proteins [21] or specific gene mutations [22, 23]. Teratomas also served as a tool in the research of skeletal muscle formation. In 1990, Muntener and coworkers investigated the interaction between neurons and muscle tissues in teratomas developing in the testes of 129/Sv mice and reported formation of neuromuscular junction (NMJ) [15]. Teratomas were also used to analyze the introduction of human artificial chromosome carrying functional gene coding dystrophin into iPSCs derived from *mdx* mice [17]. Next, within teratomas, mesoangioblast-derived iPSCs were more prone to differentiate into muscles than into other types of cells [16]. Recently, Chan and coworkers reported that PSCs differentiating within teratomas produced functional embryonic-like muscle stem cells which were able to engraft with high efficiency and regenerate serially injured muscle [24]. Thus, teratomas certainly allow to study terminal myogenic differentiation, including the formation of myoblasts, myotubes, and innervation of myofibers, i.e., analyze skeletal muscle formation within the complex in vivo environment ([15], for the review see [25]). Such model could be also useful to test the molecular network behind the “decisions” taking place during the ESC myogenic differentiation, especially during the regulation of the embryonic-fetal transition occurring during myogenesis. Thus, taking into consideration all data supporting the teratomas as a tool to test PSC potency, we decided to use it as a model allowing to determine the role of Pax7 in ESC differentiation.

During embryonic development, the Pax transcription factors are involved in the regulation of cellular distribution, specification, differentiation, and finally organogenesis [26, 27]. Pax3 and Pax7 are paralogs which contain a characteristic set of domains, including a paired domain, an octapeptide motif, and a homeodomain (for the review, see [28]). They are involved in muscle development, i.e., regulate behavior of myogenic progenitors and their entry into the program of skeletal muscle formation (reviewed in [29, 30]). Pax3 function is indispensable for migration of muscle precursors to the developing limbs [31]. Its expression is downregulated in most hindlimb muscles before birth, whereas it is maintained in the limited subpopulation of muscle-specific stem cells—satellite cells (SCs), within most forelimb and trunk muscles [32]. In contrast, Pax7 role in muscle development appears to be less critical, i.e., mice lacking this factor are characterized by reduced amount of muscle tissue but histological structure of muscles is generally normal [33, 34]. However, such muscles present significant loss or even lack of SCs [35, 36].

Pax3 and Pax7 impact the myogenic precursor cell specification and differentiation by regulating the expression of genes encoding myogenic regulatory factors (MRFs): myogenic factor 5 (Myf5), myogenic differentiation 1 (MyoD), muscle-specific regulatory factor 4 (Mrf4), and myogenin (MyoG) (for the review, see [28]). Additionally, during myogenesis, Pax3 and Pax7 control transition of myoblast differentiation from embryonic to fetal one [37]. In mouse embryo, embryonic, i.e., primary, myogenesis depends on Pax3 function and occurs between 11 and 13 days of development [37, 38]. Population of myogenic precursor cells gives rise to embryonic myoblasts which express the so-called early MRFs, i.e., MyoD, Myf5 [39], and specific markers, such as Pax3, nuclear factor of activated T cells (Nfatc4, [40]), myocyte enhancer factor 2C (Mef2c, [41]), or isoform 3 of myosin heavy chains (Myh3, [42]). Next, embryonic myoblasts differentiate to primary myotubes, which become innervated between 14 and 15 days of development ([43], reviewed in [44]). Finally, primary myotubes form slow twitching myofibers expressing the so-called late MRFs: Mrf4, MyoG, and myosin heavy chain isoforms 7 (Myh7, [40, 42]). Simultaneously, between 15 and 18 days of development, fetal, i.e., secondary, myogenesis begins. At this stage, muscle precursor cells downregulate Pax3, start to express Pax7, and differentiate into fetal myoblasts [27]. Except MyoD and Myf5, fetal myoblasts express such specific markers as nuclear factor one X (Nfix, [45]), enolase 3 (Eno3, [46]), myocyte enhancer factor 2A (Mef2a, [45]), muscle creatine kinase (Mck, [41]), and integrin subunit alpha 7 (Itga7, [47]). Secondary myotubes, which arise from fetal myoblasts, are generally smaller from adjacent primary myofibers, and their formation depends on motoneuron innervation [48]. They form fast twitching myofibers which express myosin heavy chain isoforms 2 (Myh2) and also late MRFs [42, 49].

Previous in vitro study conducted by Messina and coworkers suggested that Pax7 participates in switching myoblast differentiation from embryonic to fetal [45]. In in vitro cultured myoblasts isolated from 11.5 d.p.c. mouse embryos, Pax7 was sufficient to activate transcription of *Nfix* and thus initiate the fetal program [45]. Nfix activates the expression of fetal-specific genes: *Prkcq*—encoding protein kinase C theta (nPKC-theta), *Itga7*, and *Mef2a*. Mef2a regulates transcription of genes encoding metabolic enzymes Mck and Eno3. At the same time, the expression of the embryonic specific genes, such as *Pax3*, as well as *Myh3* and *Myh7*, and *Nfatc4*, which activate expression of *Myh7*, was repressed [45]. Transcriptional inhibition of *Nfi* gene family (including *Nfix*) affects fetal myoblast differentiation and fusion by decreasing the expression of *MyoG* and *Itga7* [45]. Consequently, less myoblasts was able to fuse, and for this reason, resulting myotubes were considerably smaller. In Pax7−/− mouse embryos, analyzed at 16.5 d.p.c., *Nfix* expression was decreased, but normal in 14-day-old Pax7−/− mouse [45]. However, the role of Pax7 as intermediary factor involved in switching from embryonic to fetal program was not fully dissected. Thus, the crucial information on Pax7 function is still missing.

In our previous studies, we analyzed the role of Pax7 in the formation of myoblasts from mouse ESCs differentiating in vitro in embryoid bodies [3] as well as in the presence of methylation reducing agent 5-azacytidine [8]. Using EBs as a differentiation model, we showed that even in the absence of functional Pax7 ESCs could form Pax3+, MyoD+, or Myf5+ myoblasts and multinucleated myotubes [3]. Next, the absence of functional Pax7 did not influence *Nfix* transcription level and fetal myoblast formation during in vitro differentiation of ESCs [3]. The major differences between Pax7+/+ and Pax7−/− cells concerned the cell cycle regulation and miRNA expression [3, 4]. Thus, our in vitro studies confirmed that the absence of functional Pax7 caused positive effect on ESC proliferation and early stages of their myogenic differentiation, as well as on the proliferation of mouse embryonic fibroblasts [4]. On the other hand, in the presence of methylation reducing agent 5-azacytidine, ESCs lacking functional Pax7 (Pax7−/− ESCs) were characterized by higher expression of mesodermal lineage-specific markers, such as platelet-derived growth factor receptor alpha (Pdgfra), and also myogenic cell markers like *Pax3*, Myf5, and MyoD [8]. We also demonstrated that in vivo Pax7−/− ESCs were able to form teratomas composed of cells and tissue originating from ecto-, endo-, and mesoderm [3]. Next, regardless of genotype, ESCs transplanted into regenerating muscle were detectable at day 7 of regeneration. Importantly, Pax7−/− ESCs more efficiently populated regenerating muscle than Pax7+/+ ESCs. Furthermore, Pax7−/− ESCs proliferated more vigorously within the regenerating muscle. Unfortunately, we were not able to localize ESCs in satellite cell niche [8]. Abovementioned studies did not concern, however, detailed analysis of the Pax7 role in in vivo differentiation of ESCs. In the current project, using teratomas, we studied the role of Pax7 at the early and advanced stages of in vivo ESC myogenic differentiation. We also determined the role of Pax7 in the innervation and development of the myofiber types. Finally, we followed the formation of satellite cells in teratomas obtained from Pax7+/+ ESCs and Pax7−/− ESCs.

## Methods

Animal studies were approved by Local Ethics Committee No. 1 in Warsaw, Poland (permit number 356/2017), according to European Union Directive on the approximation in laws, regulations, and administrative provisions of the Member States regarding protection of animals used for experimental and scientific purpose [50, 51]. All mice were raised on the premises and maintained in the 12-h light/12-h dark cycle.

### Preparation of feeder cells

Feeder cells, i.e., inactivated mouse embryonic fibroblasts (MEFs), were prepared according to Robertson [52]. Briefly, primary MEFs were isolated from 13.5-day-old embryos of F1(C57Bl6NxCBA/H) mice and cultured in DMEM (with 4.500 mg/l glucose, Gibco) supplemented with 10% heat inactivated fetal bovine serum (FBS, Gibco) and penicillin and streptomycin (5.000 units penicillin and 5 mg streptomycin/ml, Gibco). They were passaged several times and finally, after reaching confluency, inactivated by treatment with mitomycin C (10 μg/ml, Sigma-Aldrich) for 2 h. Growth-arrested MEFs were then frozen and seeded as required.

### Generation and in vitro culture of ESCs

C57Bl6N females carrying mutation in one allele of Pax7 gene were crossed with 129Sv males and genotyped as described previously [33]. ESCs were generated from blastocysts recovered from F1(C57Bl6Nx129Sv) Pax7+/− females induced to superovulate by injection of 10 IU of pregnant mare’s serum gonadotropin (PMSG, Folligon, Intervet) followed 48–52 h later by 10 IU of human chorionic gonadotropin (hCG, Folligon, Intervet) injection, allowed to mate with Pax7+/− males of the same cross. Pregnant females were sacrificed 96 h after hCG injection. ESCs were derived and characterized as described previously [3], e.g., all the lines were genotyped and karyotyped. Medium for ESC derivation was composed of KnockOut Dulbecco’s modified Eagle’s medium (KnockOut DMEM, Gibco) supplemented with 10% serum replacement (SR, Gibco) with addition of nonessential amino acids (0.1 mM, Gibco), l-glutamine (2 mM, Gibco), β-mercaptoethanol (0.1 mM, Sigma-Aldrich), penicillin and streptomycin (5.000 units penicillin and 5 mg streptomycin/ml, Gibco), murine leukemia inhibitory factor (LIF, 1000 IU/ml, ESGRO, Chemicon International), and 12.5 μM MEK1 inhibitor—PD98059 (Sigma-Aldrich). All experiments were carried out on three wild type Pax7+/+ ESC lines (B3, B5, B8) and three knock-out Pax7−/− ESC lines (B4, AI7.15, T2M4), which we described previously [3].

### In vivo differentiation of ESCs—teratoma formation

Three lines of ESC control (Pax7+/+, i.e., B3, B5, B8) and three lines of ESCs lacking functional Pax7 (Pax7−/−, i.e., B4, AI7.15, T2M4) were cultured under standard conditions to support pluripotency. After 4–5 days of culture, ESC colonies were disaggregated in 0.05% trypsin/EDTA for 3–5 min, washed once in the culture medium, and then twice in PBS. Finally, 10 × 10^6^ cells were suspended in 100 μL 0.9% NaCl and injected subcutaneously to isoflurane-anesthetized 3-month-old F1(C57Bl6Nx129Sv) males. Thirty days post ESC transplantation, if teratomas were 1 cm in diameter, then they were isolated, weighed, frozen in liquid nitrogen cooled isopentane, and then stored at − 80 °C. Infrequently, teratomas developed very small (diameter ≤ 5 mm) and poorly differentiated, such material was excluded from the analysis.

### Histological analysis

Tissue sections of 10-μm thickness were obtained from frozen teratomas using cryostat (Microm HM 505N; Microm International GmbH), air-dried, stained with Harris’s hematoxylin (Sigma-Aldrich) for 10 min and eosin Y (Sigma-Aldrich) for 5 min, and mounted in aqueous permanent mounting medium for microscopy (Dako). Histological sections from teratomas were also stained with Harris’s hematoxylin for 10 min, Gomori Trichrome (Sigma–Aldrich) for 60 min, and mounted in aqueous permanent mounting medium for microscopy (Dako) according to the manufacturer’s instructions. The histological scanner (Axio Scan Z1 Zeiss) and Gimp2 software were used to evaluate the teratoma histology and to identify the area of muscle tissue in relation to the total area of teratoma section. Five frozen sections from different parts of each teratoma were analyzed. Briefly, using Gimp2 software, muscle tissue visible within the section was contoured and filled with color. Next, the number of pixels within the colored area was counted automatically and the proportion of the “colored area” to the total surface, i.e., total number of pixels within the analyzed section, was estimated.

### Immunolocalization

Teratoma cryosections were fixed with 3% paraformaldehyde (Sigma-Aldrich) in PBS, at room temperature for 10 min, and permeabilized with 0.05% Triton-X 100 (Sigma-Aldrich) in PBS, at room temperature for 3 min. The nonspecific antibody binding sites were blocked by incubation in 3% bovine serum albumin (BSA) in PBS at room temperature for 30 min. Next, specimens were incubated in primary antibody solutions, i.e., antibodies anti: Myf5 (ab125301, Abcam; 1:100), Laminin (L9393, Sigma-Aldrich; 1:500), Laminin (L8271, Sigma-Aldrich; 1:500), skeletal muscle myosin heavy chains (skMyh, M7523, Sigma-Aldrich; 1:100), embryonic isoforms of myosin heavy chains (Myh3, F1.652, DSHB; 1:10), slow isoforms of myosin heavy chains (Myh7, BA-D5, DSHB; 1:10), fast isoforms of myosin heavy chains IIa (Myh2a, 2F7, DSHB; 1:10), fast isoforms of myosin heavy chains IIb (Myh2b, BF-F3, DSHB, 1:50), neurofilament (NF-M, 2H3, DSHB, 1:50), synaptophysin 1 (101,004, Synaptic Systems, 1:50), and AChR inhibitor fasciculin-II (F-650, Alomone Lab, Rhodamine conjugated, 7 μg/ml) diluted in 0.5% BSA in PBS, at 4 °C overnight. Afterwards, teratoma cryosections were incubated with appropriate secondary antibody conjugated with Alexa 488 (Molecular Probes), Alexa 594 (Molecular Probes), or Alexa 633 (Molecular Probes) diluted 1:200 in 0.5% BSA in PBS at room temperature for 2 h. To visualize the nuclei, specimens were incubated in DRAQ5 (Biostatus Limited) diluted 1:500 in PBS for 5 min. Finally, the specimens were mounted with fluorescent mounting medium (DakoCytomation). Specificity of primary antibodies was confirmed by incubation of samples with secondary antibodies only. The specimens were analyzed using Axio Observer Z1 scanning confocal microscope (Zeiss) equipped with LSM 700 software (Zeiss). To analyze myogenic cell localization and myofiber formation, five frozen sections from different parts of each teratoma were examined. The number of Myf5+ cells was counted using “ImageJ cell counter.” Gimp2 software was used to evaluate the surface of skMyh. Briefly, visualized skMyh was contoured and filled with color. Next, the number of pixels within the colored area was counted automatically and the percentage of the “colored area” to the total surface, i.e., total number of pixels within the analyzed section, was estimated.

### Analysis of different myofiber types’ representation

Using teratoma cryosections, immunolocalization of myofibers expressing embryonic, slow, or fast myosin heavy chains (Myh3, Myh7, Myh2a, or Myh2b) was performed (immunolocalization procedure described above). The histological scanner (Axio Scan Z1 Zeiss, available at the Department of Immunology, Medical University of Warsaw) and Gimp2 software were used to evaluate the area occupied by embryonic, slow, and fast myofibers. The area of myofibers expressing Myh3, Myh7, Myh2a, or Myh2b was contoured and filled with color. Next, the number of pixels within the colored area was counted automatically and the percentage of the “colored area” in relation to the total area of teratoma section was estimated. Each analysis was performed at least in three independent biological repeats.

### Analysis of neuromuscular junctions

Teratoma cryosections were prepared using standard procedure. To visualize myofibers, we used skeletal muscle myosin heavy chain antibody (skMyh, M7523, Sigma-Aldrich; 1:100); to visualize the neuromuscular junctions, we used α-Bungarotoxin conjugated with Alexa 488 (5 μg/ml, B13422, Molecular Probes) added to the secondary antibody at the dilution 1:200. The fluorescence of α-Bungarotoxin bound to acetylcholine receptor was analyzed using Axio Observer Z1 scanning confocal microscope (Zeiss) equipped with LSM 700 software. Figures were assembled using Adobe Photoshop CS6 Extended. To estimate and compare dimensions of NMJ-positive (NMJ^pos^) and NMJ-negative (NMJ^neg^) myofibers, we analyzed 3 fields of view of 3 teratoma cryosections for each Pax7+/+ and Pax7−/− ESC line. At first, we counted the area of each myofiber using Gimp2 software. The obtained area of myofiber was compared to the circle with the same area (*A*). Using the equation for the area of a circle (*A* = *πR*^2^), we estimate the radius of the circle (*R* = √*A*/*π*). Then knowing the exact scale on the photo and formula on diameter (*D* = 2*R*), we estimated the diameter of each circle, i.e., myofiber.

### 3D NMJ analysis—tissue clearing (iDISCO) and imaging

One cubic centimeter teratoma blocks were stained and cleared with modified iDISCO+ protocol [53]. Samples were dehydrated in increasing concentrations of methanol and further bleached with H_2_O_2_ solution (1:5 of 30% H_2_O_2_ in methanol). After rehydration, samples were stained with α-Bungarotoxin conjugated with Alexa 647 (ThermoFisher, B35450; 1:1000) for 3 days. Next, teratoma blocks were washed with PtwH solution (PBS/0.2% Tween-20 with 10 μg/ml heparin) and dehydrated in increasing concentrations of methanol. Methanol was further washed out with dichloromethane (DCM) at first using solution of 1:2 methanol in DCM followed by two washes in pure DCM. Finally, samples were placed for 1 week in dibenzyl ether (DBE). Cleared samples were imaged with home-built light-sheet microscope [54, 55] equipped with immersion objective LaVisionBioTec 4x NA 0,5, WD 6 mm and camera Hamamatsu Orca-Flash4.0. Laser 638 nm was used for excitation of fluorophores. Data were collected with 4-μm Z step and resolution in XY 1.45 × 1.45. Single plane was acquired from sequence of photos 800 × 1500 μm. TeraStitcher v 1.10.4 was used for stitching of tiles [56]. Videos were prepared in Imaris (Bitplane Inc. Imaris v 9.1) for surface generation; background was settled for values below 150 in grayscale.

### RNA isolation and qPCR

mRNA was isolated from teratomas, using mirVana Isolation Kit (Thermo Fisher Scientific). mRNA analysis reverse transcription was performed using 1 μg total RNA and RevertAid First Strand cDNA Synthesis Kit (Thermo Fisher Scientific) according to the manufacturer’s instruction. qPCR was performed using specific TaqMan® probes: Mm02019550_s1 (*Nanog*), Mm00435493_m1 (*Pax3*), Mm00435125_m1 (*Myf5*), Mm00440387_m1 (*Myod1*), Mm00446194_m1 (*MyoG*), Mm00483191_m1 (*Cdh15*), Mm01318252_m1 (*T* encoding Brachyury), Mm00477791_m1 (*Nfix*), Mm00468267_m1 (*Eno3*), Mm01321487_m1 (*Mck*), Mm00434400_m1 (*Itga7*), Mm01318991_m1 (*Mef2a*), Mm00452375_m1 (*Nfatc4*), Mm01332463_m1 (*Myh3*), Mm01332564_m1 (*Myh2*), Mm01319006_g1 (*Myh7*), Mm01332541_m1 (*Myh4*), Mm01226102_m1 (*Lama1*), Mm00493080_m1 (*Lamb2*), Mm01193660_m1 (*Lama4*), Mm01222010_g1 (*Lama5*), Mm01248771_m1 (*RbFox3*), Mm00446859_m1 (*Otx2*), Mm01205647_g1 (*Actb*), and the TaqMan Gene Expression Master Mix (Thermo Fisher Scientific). Data were normalized against *Actb*. Data were also standardized against expression observed in mouse embryos at day 13.5. All qPCRs were performed on LightCycler96 instrument (Roche). Amplification curves were analyzed using LightCycler 96 SW1.1 software (Roche) for determination of Ct. 2^–ΔΔCT^ analysis was performed according to Livak and Schmittgen [57].

### Western blotting

Teratomas were homogenized and lysed in RIPA buffer containing protease inhibitors (Complete, Roche), and obtained lysates were kept on ice for 30 min, centrifuged, and stored at − 80 °C. Protein concentration was determined by using Bradford’s assay (Sigma-Aldrich). Twenty microgram protein was subjected to 10% SDS-PAGE and Western blot analysis and probed with antibodies against Pax3 (ARP32446, Aviva; 1:1000), Myf5 (SAB 4501943, Sigma-Aldrich; 1:1000), MyoD (Ab203383, Abcam; 1:1000), Myogenin (sc-576, Santa Cruz Biotechnology; 1:1000), Mck (SAB4500267, Sigma-Aldrich; 1:1000), M-cadherin (SAB4500040, Sigma; 1:1000), or Hsp90 (TA500494, OriGene Technologies). As secondary antibodies, goat anti-rabbit HRP conjugate (170-6515, Bio-Rad) was used, followed by chemiluminescence detection. Films (Amersham Hyperfilm ECL (GE Healthcare)) exposed on membranes were photographed, and optical density of resulting bands was measured using GelDocXR+ (Bio-Rad) with ImageLab software.

### Data analysis

Sample size was calculated based on GPower 3.1.9.4 to ensure adequate power of the test. Data was analyzed and visualized using Prism version 7.0 (GraphPad Software, Inc.). Experimental repeats (*n* values) are specified in figure legends. At first, data was tested for normal distribution using the Shapiro-Wilk test. To compare the respective variances of two groups, we applied Fisher’s *F* test. Student’s unpaired *t* test (two-tailed) for comparisons between two groups was used when normal distribution could be assumed. If data was characterized by non-normal distribution, we performed normalization of the data by transformation in log values. After normalization, distribution of the data was checked and then we compare them using Student’s unpaired *t* test (two-tailed). Values of *p* < 0.05 were considered statistically significant [*p* < 0.05 (*), *p* < 0.01 (**), *p* < 0.001 (***), and *p* < 0.0001 (****)]. One-way ANOVA and post hoc Tukey’s multiple comparison tests were used to analyze differences between various groups of myofibers in teratomas. The level of significance was set at *p* < 0.05.

## Results

### Myogenic differentiation of Pax7+/+ and Pax7−/− ESCs in teratomas

**Fig. 1:**
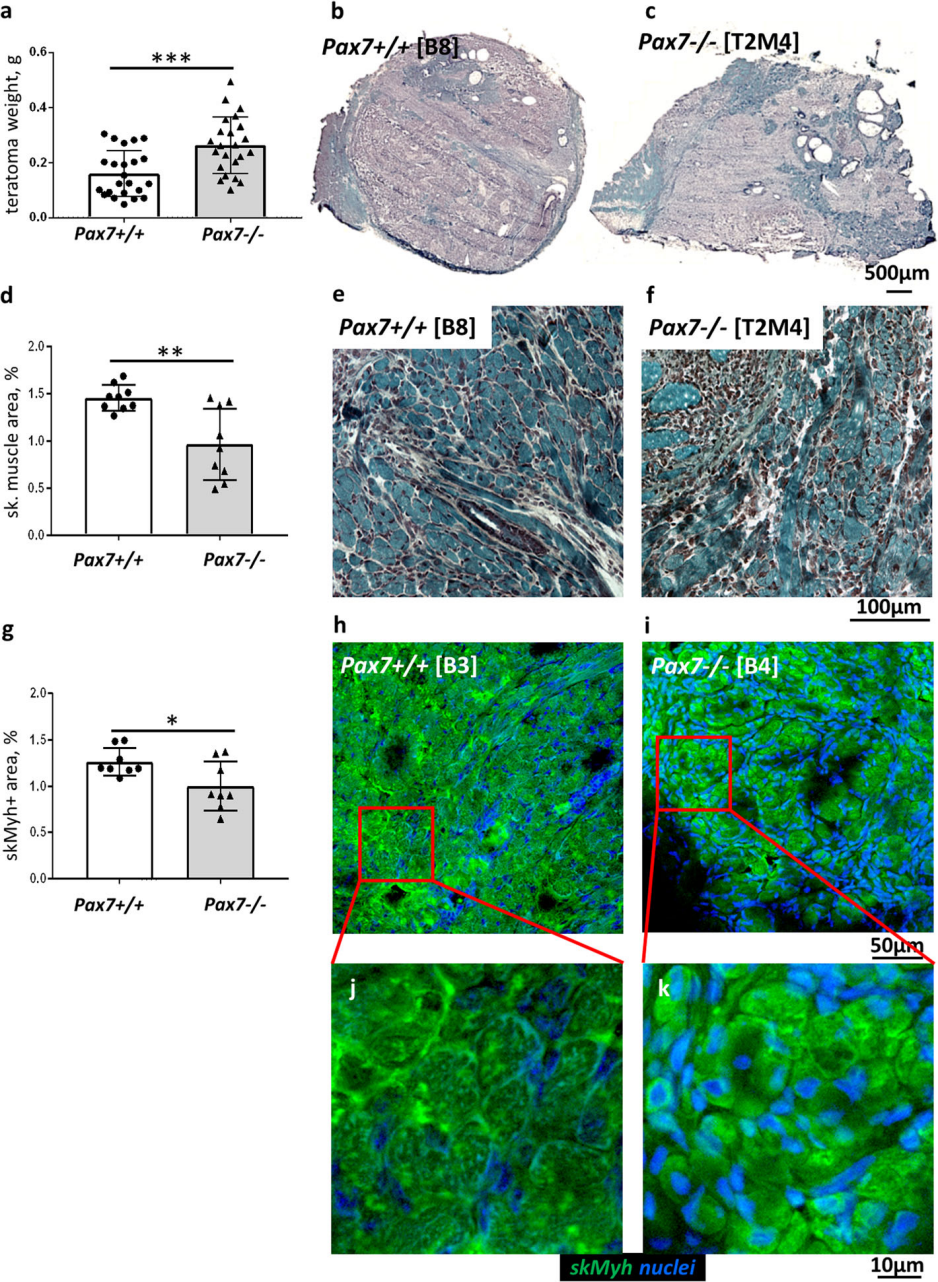
Muscle tissue in Pax7+/+ and Pax7−/− ESC teratomas. **a** Weight of Pax7+/+ and Pax7−/− teratomas (for each genotype *n* = 23). **b**, **c** Representative cross sections of entire teratomas stained with Harris hematoxylin and Gomori Trichrome dye. Scale bar 500 μm. **d** Calculation of the area occupied by skeletal muscle tissue in teratomas (for each genotype *n* = 9). **e**, **f** Morphology of skeletal muscle tissue (blue) stained with Harris hematoxylin and Gomori Trichrome dye. Scale bar 100 μm. **g** Calculation of the area of cells expressing skeletal muscle myosin heavy chains (skMyh) (for each genotype *n* = 9). **h**, **i** Immunolocalization of skMyh (green) and localization of nuclei (blue). Scale bar 50 μm. **j**, **k** Inserts—magnification of myofibers expressing skMyh in teratomas. Scale bar 10 μm. White bars—values for teratomas derived from Pax7+/+ ESCs, gray bars—values for teratomas derived from Pax7−/− ESCs. Data characterized by non-normal distribution (**a**, **d**, **g**) was normalized. Data are presented as mean ± SD. Stars represent results of Student’s unpaired two-tailed *t* test: *p < 0.05; **p < 0.01; ***p < 0.001

We used mouse ESCs lacking functional Pax7 to dissect the role of this factor at subsequent stages of myogenic differentiation, i.e., up to the time when mature, innervated myofibers are formed. These cells were previously generated and characterized by us [3, 4, 8]. Currently, using the teratoma model, we analyzed differentiation of wild type, i.e., Pax7+/+ ESCs (ESC lines B3, B5, B8), and ESCs lacking functional *Pax7* gene, i.e., Pax7−/− ESCs (ESC lines B4, AI7.15, T2M4). We assessed the weight and histological structure of Pax7+/+ and Pax7−/− teratomas (Fig. 1a–c). The weight of Pax7−/− teratomas was approximately two times higher than that of Pax7+/+ teratomas (Fig. 1a), what supported our earlier observations showing that lack of functional Pax7 increases the proliferation of differentiating ESCs [4]. Our previous [3] and current histological analyses revealed that Pax7+/+ and Pax7−/− teratomas contained cells and tissues of all three germ layers, including skeletal muscle (Fig. 1b, c). Importantly, in Pax7−/− teratomas, the area occupied by skeletal muscle was significantly smaller in comparison to Pax7+/+ teratomas (28.9% vs 12.3%) (Fig. 1d). Interestingly, smaller myotubes and myofibers dominated in the Pax7−/− teratomas (Fig. 1e, f). Analysis of skeletal muscle myosin heavy chain (skMyh) localization showed that in Pax7−/− teratomas the skMyh+ area was reduced in comparison to Pax7+/+ teratomas (Fig. 1g–i). We also visualized immature myofibers characterized by smaller diameter, centrally located nuclei and less organized contractile bundles in Pax7−/− teratoma muscles (Fig. 1j, k).

#### Molecular characteristic of skeletal muscles present within teratomas

**Fig. 2:**
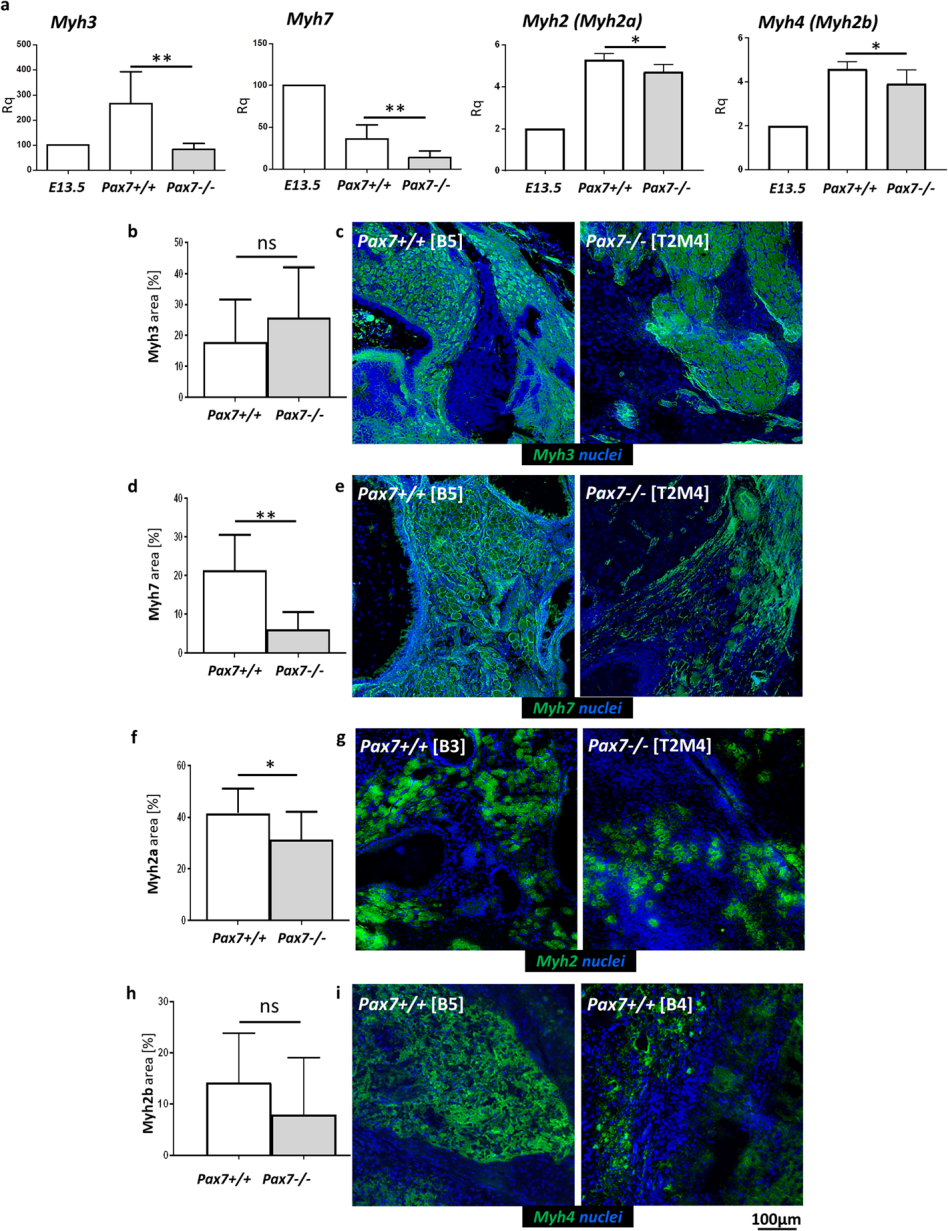
Analysis of the type of myofiber formation in teratomas obtained from Pax7+/+ and Pax7−/− ESCs. **a** Expression of mRNAs encoding four isoforms of skeletal myosin heavy chains: *Myh3*, *Myh7*, *Myh2*, and *Myh4* (for each genotype *n* = 6 or *n* = 7). Expression was related to the levels observed in 13.5 d.p.c. mouse embryo (E13.5; *n* = 3), and normalized to mRNA encoding β-actin (*Actb*). **b** Area occupied by myofibers expressing embryonic isoform of myosin heavy chains (Myh3) (for each genotype *n* = 6). **c** Localization of embryonic (Myh3, green) myofibers; localization of nuclei (blue). **d** Area occupied by myofibers expressing slow isoform of myosin heavy chains (Myh7) (for each genotype *n* = 6). **e** Localization of slow twitching (Myh7, green) myofibers; localization of nuclei (blue). **f** Area occupied by myofibers expressing fast isoform of myosin heavy chains (Myh2a) (for each genotype *n* = 9). **g** Localization of fast twitching (Myh2a, green) myofibers; localization of nuclei (blue). **h** Area occupied by myofibers expressing fast isoform of myosin heavy chains (Myh2b) (for each genotype *n* = 6). **i** Localization of fast twitching (Myh2b, green) myofibers; localization of nuclei (blue). Scale bar 100 μm. White bars—values for Pax7+/+ teratomas, gray bars—values for Pax7−/− teratomas. Data characterized by non-normal distribution (**a**, Myh2, Myh4) was normalized. Data are presented as mean ± SD. Statistically nonsignificant data was marked as “ns”. Stars represent results of Student’s unpaired two-tailed *t* test: *p < 0.05; **p < 0.01

To test how Pax7 influences expression of different isoforms of myosin heavy chains (Myh), we examined the levels of the following mRNAs: *Myh3* (gene coding embryonic isoform of muscle myosin heavy chain), *Myh7* (coding type I/slow isoform of muscle myosin heavy chain), *Myh2* (coding fast isoform IIa of muscle myosin heavy chain), and *Myh4* (coding fast isoform IIb of muscle myosin heavy chains) (Fig. 2a). To standardize the results, we referred the expression levels of analyzed genes to the expression level observed in 13.5 d.p.c. mouse. The levels of mRNAs coding myosin heavy chain isoforms were significantly lower in Pax7−/− teratomas. Next, we analyzed the area occupied by cells and myofibers expressing myosin heavy chains Myh3, Myh7, Myh2a, and Myh2b (Fig. 2b–i). The proportion of Myh3 and Myh2b myofibers did not differ between Pax7+/+ and Pax7−/− teratomas (Fig. 2b, c, h, i). However, the area of Myh7 and Myh2a myofibers was significantly lower in Pax7−/− teratomas (Fig. 2d–g).

#### Gene expression profiling in Pax7+/+ and Pax7−/− ESC teratomas

**Fig. 3:**
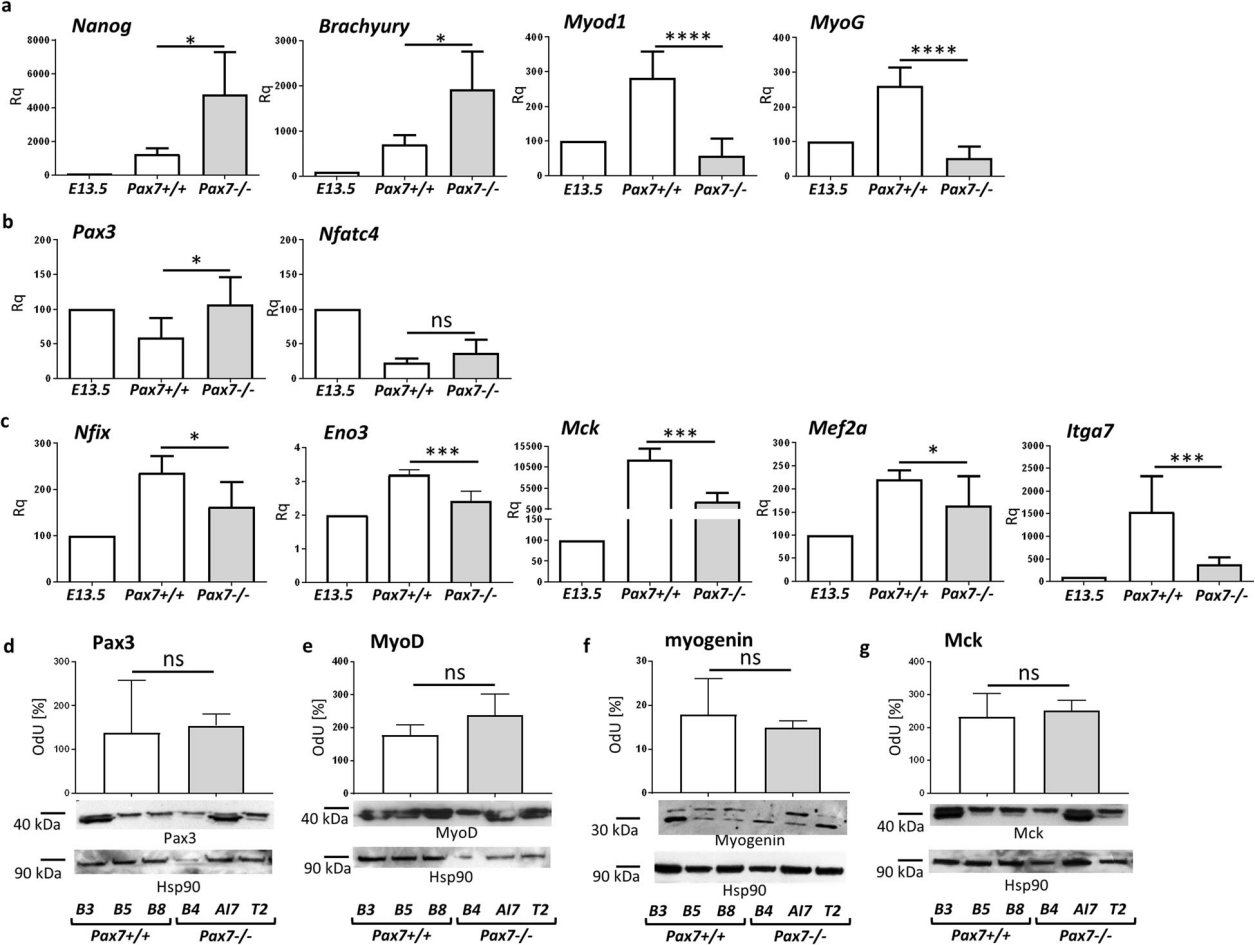
Selected gene expression in teratomas obtained from Pax7+/+ and Pax7−/− ESCs. **a** Expression of mRNAs encoding pluripotency marker (*Nanog*), marker of primitive streak—mesodermal cell marker (*Brachyury*), and markers of myogenic cells (*Myod1*, *MyoG*) (for each genotype *n* = 7). **b** Expression of mRNAs encoding embryonic myoblast markers (*Pax3*, *Nfatc4*) (for each genotype *n* = 7). **c** Expression of mRNAs encoding fetal myoblast markers (*Nfix*, *Eno3*, *Mck*, *Mef2a*, *Itga7*) (for each genotype *n* = 5 to *n* = 9). Expression was related to the levels observed in 13.5 d.p.c. mouse embryo (E13.5; *n* = 3), and normalized to mRNA encoding β-actin (*Actb*). **d**–**g** Western blot analysis of Pax3, MyoD, myogenin, and Mck in teratomas obtained from three Pax7+/+ ESC lines and three Pax7−/− ESC lines (for each genotype *n* = 3). Hsp90 level was used as a loading control. Graph represents optical density of Pax3, MyoD, Myogenin, and Mck bands compared to density of corresponding Hsp90 bands (optical density of Hsp90 was accounted as 100%, OdU optical density, arbitrary units). White bar—values for Pax7+/+ teratomas, gray bar—values for Pax7−/− teratomas. Data characterized by non-normal distribution (**c**, *Eno3*) was normalized. Data are presented as mean ± SD. Stars represent results of Student’s unpaired two-tailed *t* test: *p < 0.05; **p < 0.01; ***p < 0.001; ****p < 0.0001

Next, we focused at the expression of mRNAs encoding pluripotency, mesoderm formation, and myogenic factors. *Nanog* mRNA expression, as well as the level of mesoderm marker *T*, encoding Brachyury, was much higher in Pax7−/− teratomas in comparison to Pax7+/+ teratomas (Fig. 3a). However, *Myod1* and *MyoG* expression was significantly lower in Pax7−/− teratomas (Fig. 3a). To distinguish between primary (embryonic) and secondary (fetal) myogenesis, we assessed the expression of genes coding markers of primary, i.e., Pax3 and Nfatc4, and secondary (fetal) myoblasts, i.e., Nfix, Eno3, Mck, Mef2a, and Itga7. The level of mRNAs encoding Pax3 was significantly higher (Fig. 3b), while the expression of *Nfix*, *Eno3*, *Mck*, *Mef2a*, and *Itga7* was significantly lower in Pax7−/− teratomas, as compared to Pax7+/+ teratomas (Fig. 3c). Next, we analyzed the Pax3, MyoD, Myogenin, and MCK protein levels (Fig. 3d–g). We showed data for Pax3, MyoD, Myogenin, and MCK (samples were obtained from teratomas of 3 independent ESC lines for each genotype). Pax3 level was slightly higher and Myogenin level was slightly lower in Pax7−/− teratomas than in Pax7+/+ teratomas (Fig. 3d, f). Levels of MyoD and Mck did not differ between both types of teratomas (Fig. 3e, g). Together, these findings show that cells at earlier stages of myogenic differentiation dominate in Pax7−/− teratomas, as compared to Pax7+/+ teratomas. Thus, it suggests that in the absence of functional Pax7 myogenesis lags behind, i.e., the expression of mesoderm and embryonic myoblast markers is upregulated.

#### Satellite cells and their niche in Pax7+/+ and Pax7−/− skeletal muscle in teratomas

**Fig. 4:**
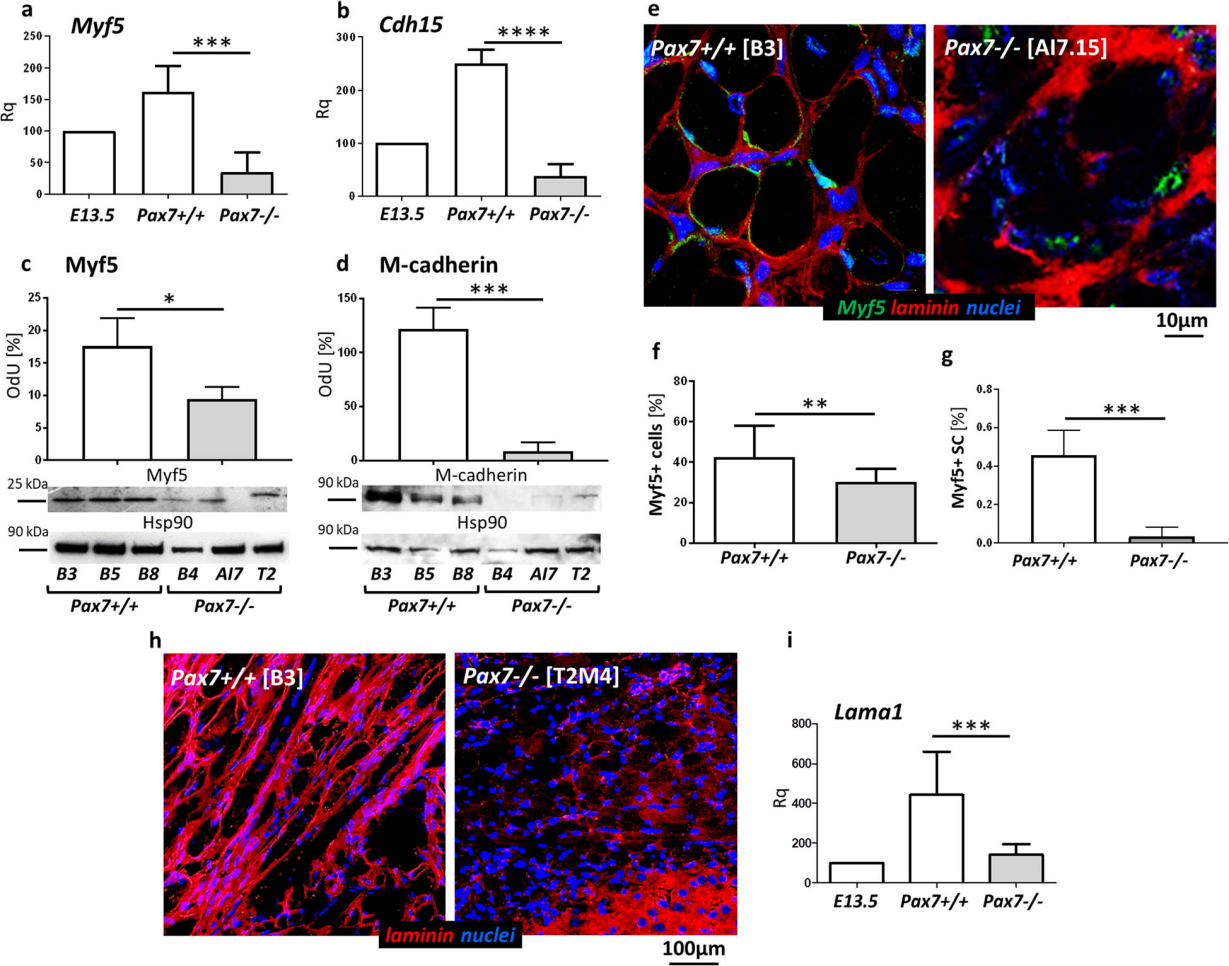
Analysis of the satellite cell population in teratomas obtained from Pax7+/+ and Pax7−/− ESCs. **a**, **b** Expression of mRNAs encoding satellite cell markers (*Myf5*, *Cdh15*) (for each genotype *n* = 6). Expression was related to the levels observed in 13.5 d.p.c. mouse embryo (E13.5; *n* = 3), and normalized to mRNA encoding β-actin (*Actb*). **c**, **d** Western blot analysis of Myf5 (for each genotype *n* = 3) and M-cadherin levels (for each genotype *n* = 3) in teratomas obtained from three Pax7+/+ ESC lines and three Pax7−/− ESC lines. Hsp90 level was used as a loading control. Graph represents optical density of Myf5 and M-cadherin bands compared to density of Hsp90 bands (optical density of Hsp90 was accounted as 100%, OdU optical density, arbitrary units). **e** Immunolocalization of Myf5+ satellite cells (green), laminin (red), and nuclei (blue) in teratoma muscles. Scale bar 10 μm. **f** Percentage of the myogenic cells (Myf5+ cells) in teratomas (for each genotype *n* = 16). **g** Calculation of the satellite cells (SC) expressing Myf5 in teratoma muscles (for each genotype *n* = 4). **h** Immunolocalization of laminin (red) and nuclei (blue) in teratoma muscles. Scale bar 100 μm. **i** Expression of *Lama1* (coding laminin alpha 1) (for each genotype *n* = 9). Expression was related to the levels observed in 13.5 d.p.c. mouse embryo (E13.5, *n* = 3), accounted as 100%, and normalized to mRNA encoding β-actin (*Actb*). White bars—values for teratomas obtained from Pax7+/+ ESCs, gray bars—values for teratomas obtained from Pax7−/− ESCs. Data characterized by non-normal distribution (**g**) was normalized. Data are presented as mean ± SD. Stars represent results of Student’s unpaired two-tailed *t* test: *p < 0.05; **p < 0.01; ***p < 0.001; ****p < 0.0001

As shown in multiple analyses of various mouse models, Pax7 is crucial for the postnatal maintenance of the population of muscle stem cells, i.e., satellite cells [32, 36, 58, 59]. To test whether Pax7 plays the role in the formation and maintenance of satellite cells in the muscles formed in vivo from differentiating ESCs, we estimated the expression of mRNAs encoding such satellite cell markers as *Myf5* and *Cdh15* (encoding M-cadherin). Since we analyzed Pax7−/− ESCs, we could not use Pax7 as a satellite cell marker. *Myf5* and *Cdh15* expression was significantly lower in Pax7−/− teratomas (Fig. 4a, b). Western blot analysis revealed that Myf5 and M-cadherin protein levels were also significantly reduced in Pax7−/− teratomas (Fig. 4c, d). Immunolocalization of Myf5+ cells showed that the total number of these cells was significantly lower in Pax7−/− teratomas (Fig. 4e, f). Next, the proportion of Myf5+ cells localized within the satellite cell niche, calculated as a the percentage of all nuclei in the muscle tissue area, was significantly lower in Pax7−/− teratomas in comparison to Pax7+/+ teratomas (Fig. 4e, g). Interestingly, immunolocalization of laminin, i.e., the main component of skeletal muscle basal lamina, showed disturbances in the arrangement of this protein in Pax7−/− teratomas (Fig. 4h). Additionally, Pax7−/− teratomas were characterized by reduced expression of *Lama1* gene coding laminin subunit alpha 1 (Fig. 4i).

#### Neuromuscular junctions in skeletal muscles of Pax7+/+ and Pax7−/− ESC teratomas

**Fig. 5:**
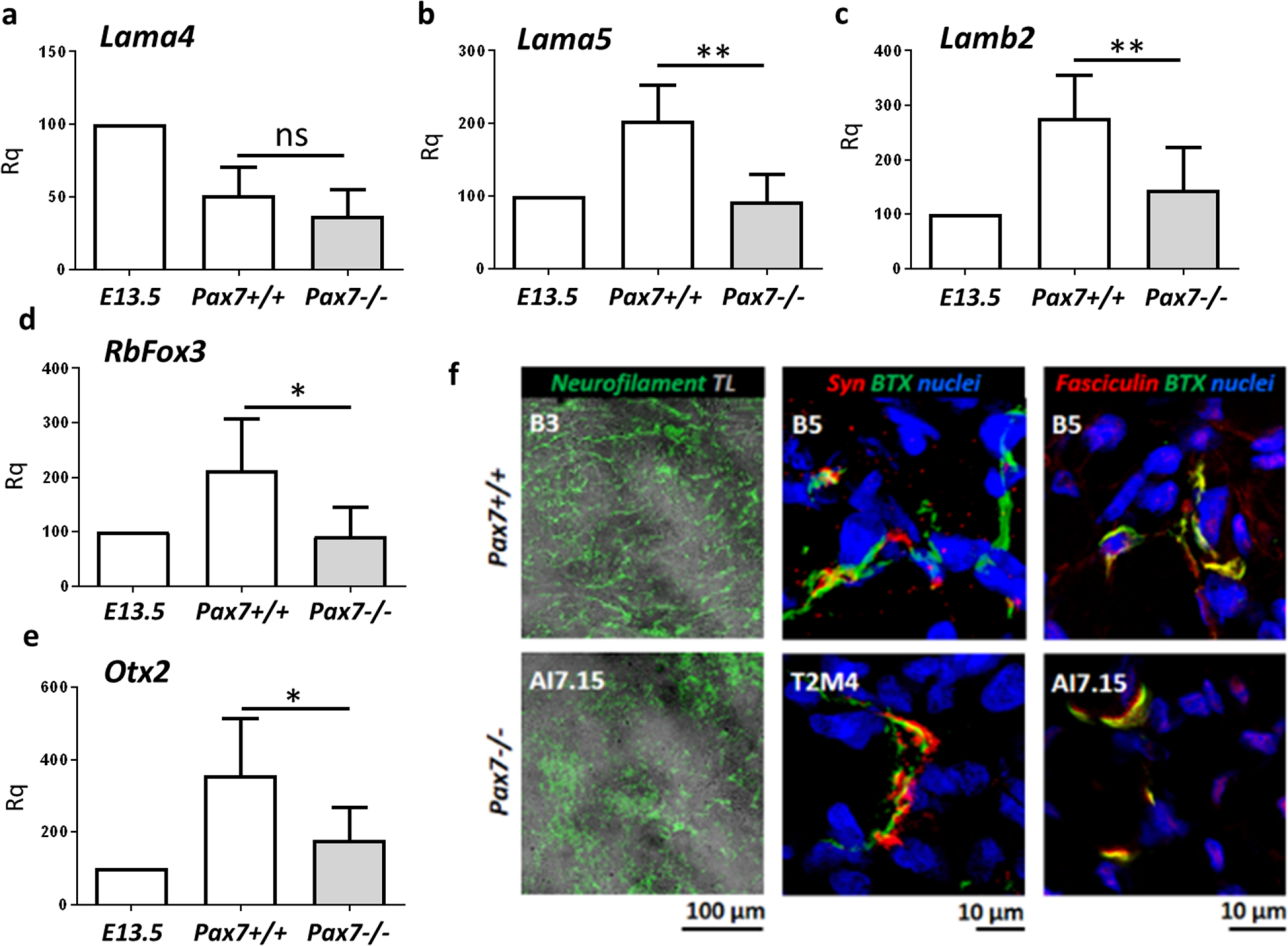
Analysis of basal lamina and NMJ functionality in Pax7+/+ and Pax7−/− teratomas. **a**–**c** Expression of mRNAs encoding *Lama4*, *Lama5*, and *Lamb2* (for each genotype *n* = 8 to 6). **d**, **e** Expression of mRNAs encoding neuronal markers *RbFox3* and *Otx2* (for each genotype *n* = 6 to *n* = 7). **f** Immunolocalization of neurofilament (green, left column, TL transmitted light), synaptophysin (red, middle column; BTX—green; nuclei—blue), and Fasciculin (red, right column; BTX—green; nuclei—blue) in teratoma muscles. Scale bar 10 or 100 μm, as indicated. White bars—values for teratomas obtained from Pax7+/+ ESCs, gray bars—values for teratomas obtained from Pax7−/− ESCs. Data are presented as mean ± SD. Stars symbolize results of Student’s unpaired two-tailed *t* test: *p < 0.05; **p < 0.01

Pax7 was shown to play a role in the development of the central nervous system [29], and Pax7-null mice are characterized by many neurological disorders [33]. Transmission of impulses from motoneurons to myofibers takes place in highly specialized chemical synapses called NMJs. Knowing that laminins are crucial for NMJ formation and clustering of postsynaptic acetylcholine receptor (AChR) [60–62] and that Pax7 influence laminin synthesis, we analyzed the expression of genes coding laminins involved in the formation of NMJs and innervation of myofibers. To test whether impaired formation of NMJs in Pax7−/− teratomas is associated with specific laminins, we estimated the expression of mRNAs encoding laminin alpha 4 (*Lama4*), laminin alpha 5 (*Lama5*), and laminin beta 2 (*Lamb2*) subunits. *Lama4* expression was on the same level in both types of teratomas (Fig. 5a), whereas *Lama5* and *Lamb2* expression was significantly lower in Pax7−/− teratomas (Fig. 5b, c). Importantly, *Lamb2* and *Lama5* have been previously established as critical for proper NMJ formation [63, 64]. Additionally, the lack of functional Pax7 resulted in the lower expression of genes that play an important role in neuronal differentiation, i.e., *RbFox3* and *Otx* (Fig. 5d, e). To visualize muscle postsynaptic machinery, we stained teratoma cryosections with fluorescently labeled α-Bungarotoxin (BTX), which binds with high affinity to postsynaptic acetylcholine receptors (AChRs) and observed synaptic-like staining on myofibers (Fig. 5f). To ensure that these are indeed NMJs, we stained tissues for various synaptic markers: neurofilament and synaptophysin to visualize nerve terminals as well as synaptic cleft component acetylcholine esterase (AChE, visualized with fluorescently labeled fasciculin toxin). Analysis of neurofilament immunoreactivity at low magnification showed that in Pax7−/− teratomas this protein was located in separated clusters while in Pax7+/+ teratomas neurofilament formed long bundles amid myofibers (Fig. 5f, left column). This suggests different organization of neuronal processes in control and Pax7−/− teratomas. High-magnification images revealed that the BTX-positive areas contain also synaptophysin (Fig. 5f, middle column) immunoreactivity and high concentration of fasciculin (Fig. 5f, right column). Thus, the observed BTX-positive structures are neuromuscular junctions containing presynaptic and postsynaptic specializations as well as NMJ-specific synaptic cleft protein AChE (Fig. 5f, middle and right column).

**Fig. 6:**
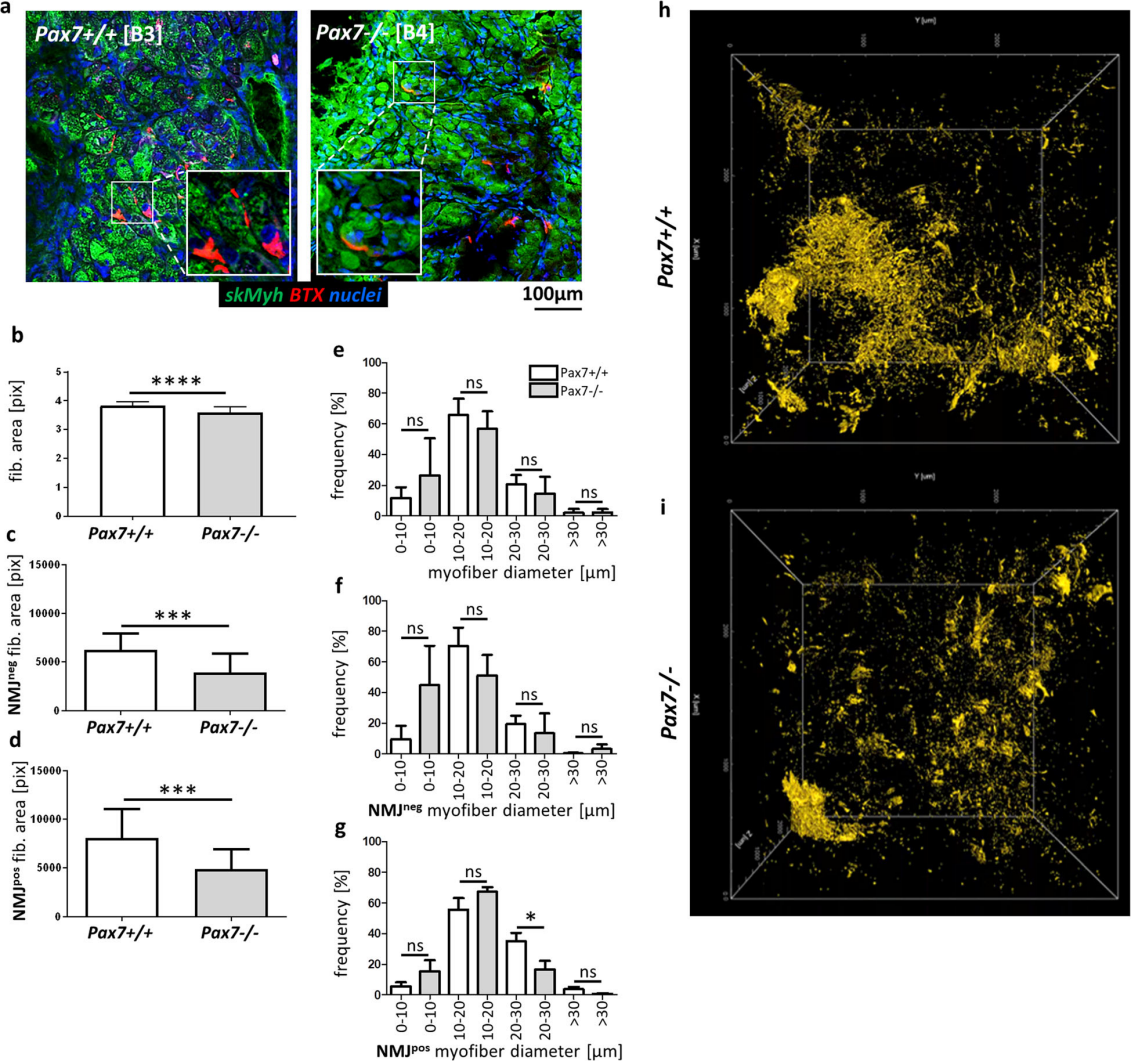
Analysis of NMJs in Pax7+/+ and Pax7−/− teratomas. **a** Immunolocalization of NMJs (BTX, red), skMyh (green), and localization of nuclei (blue) in teratoma myofibers. Scale bar 100 μm. Inserts—magnification of NMJs in teratoma muscles. **b** Calculation of the area of myofibers in teratomas (for each genotype *n* = 39). **c** Calculation of the area of NMJ^neg^ myofibers in teratomas (for each genotype *n* = 20). **d** Calculation of the area of NMJ^pos^ myofibers (for each genotype *n* = 19). **e** Analysis of the frequency of myofibers of various diameter (for each genotype *n* = 6). **f** Analysis of the frequency of NMJ^neg^ myofibers of various diameter (for each genotype *n* = 3). **g** Analysis of the frequency of NMJ^pos^ myofibers of various diameter (for each genotype *n* = 3). **h**, **i** Whole volume analysis of NMJ organization in Pax7+/+ (**h**) and Pax7−/− (**i**) teratoma samples prepared with the iDISCO method, images are single frames from a 3D visualization movie, BTX—yellow (for each genotype *n* = 1). White bars—mean values for teratomas obtained from Pax7+/+ ESCs, gray bars—mean values for teratomas obtained from Pax7−/− ESCs. Data are presented as mean ± SD. Data characterized by non-normal distribution (**b**) was normalized. **b**–**d** Stars symbolize results of Student’s unpaired two-tailed *t* test: ***p < 0.001; ****p < 0.0001. **e**–**g** Star symbolizes result of one-way ANOVA and post hoc Tukey’s multiple comparisons test. The level of significance was set at *p* < 0.05

Myofibers and NMJ were also analyzed in control and Pax7−/− teratoma sections using an anti-skMyh antibody and BTX (Fig. 6a). Teratoma muscle tissue is relatively poorly organized as a consequence of more stochastic differentiation. Accordingly, NMJs appeared to localize randomly throughout muscles. Analysis of multiple sections of Pax7+/+ and Pax7−/− teratomas allowed us to precisely investigate the area of NMJ-positive (NMJ^pos^) and NMJ-negative (NMJ^neg^) myofibers and their diameters. We showed that myofibers in Pax7−/− teratomas were smaller (Fig. 6b). Smaller myofibers were observed in case of both—NMJ^neg^ as well as NMJ^pos^ fibers in Pax7−/− teratomas (Fig. 6c, d). The differences between Pax7−/− and Pax7+/+ teratomas in fiber area were, however, greater in the case of NMJ^pos^ fibers (Fig. 6e–g). Analysis of AChR (BTX-positive structures) localization in samples prepared with the iDISCO method confirmed that the organization of NMJs in Pax7−/− teratomas was different as compared to those derived from Pax7+/+ ESCs, i.e., in Pax7−/− teratomas, NMJ organization was impaired and the amount of AChRs was reduced in comparison to Pax7+/+ teratomas (Fig. 6h, i; Additional Movie 1 [Pax7+/+] and Additional Movie 2 [Pax7−/−]).

## Discussion

In the current study, we focused at the role of Pax7 in the regulation of early and advanced stages of myogenic ESC differentiation in vivo. We put special emphasis on the formation of mature skeletal myofibers and satellite cells and neuromuscular junctions. Using teratoma assay, we addressed the question whether Pax7 acts as a “switch” allowing transition from embryonic to fetal myogenesis and whether it is necessary for the maturation of skeletal myofibers.

Our previous studies showed that in in vitro culture ESC Pax7−/− are more prone to differentiate into myoblasts [3, 8]. This conclusion resulted from the analysis of Pax7 role during the differentiation of ESCs cultured in vitro in so-called embryonic bodies (EBs) and their outgrowths (EBOs) [3], as well as in the presence of the demethylating agent 5-AzaC [8]. Using EBs as a differentiation model, it has been shown that even in the absence of functional Pax7, ESCs can form myoblasts expressing *Pax3*, *MyoD1*, and *Myf5*, and differentiate into myotubes. Pax7−/− ESCs cultured in medium containing, among others, 5-AzaC, were characterized by higher expression of mesoderm markers, i.e., *T* encoding Brachyury, *Pdgfrα*, as well as myogenic markers, i.e., *Pax3*, *Myf5*, and *MyoD1* [8]. These results allowed us to postulate that the lack of functional Pax7 may make ESCs more prone to myogenic differentiation. However, it should be remembered that these studies were conducted in vitro and focused only at the early stages of differentiation. They did not document what changes are caused by the lack of Pax7 at the late stages of myogenesis.

In all analyzed teratomas, regardless of genotype, the skeletal muscle tissue was formed. However, the area occupied by cells and myotubes expressing skeletal muscle myosin heavy chains was significantly lower in teratomas derived from Pax7−/− ESCs, as compared to control. Such phenotype indicates that Pax7−/− ESCs possess limited myogenic potential in vivo what may result in reduced ability to form mature myofibers. This observation is consistent with the results showing that mice lacking Pax7 are characterized by reduced muscle mass [33]. Moreover, histological analysis of Pax7−/− teratomas has shown the presence of immature myofibers, i.e., smaller myofibers with centrally located nuclei. Importantly, we did not observe well-organized myofibrils in such myofibers. Myofibrils present in Pax7−/− teratomas were similar to that observed in immature myofibers formed after prepubertal satellite cell ablation [65]. Furthermore, Pax7−/− teratomas were characterized by lower expression of genes coding proteins necessary for proper formation of myofiber structure, i.e., *Myh3*, Myh7, *Myh4*, and *Myh2*. Such “immature” structure of Pax7−/− myofibers was not previously reported in studies on Pax7 role during skeletal muscle development. Thus, we found that Pax7−/− ESCs showed limited potential to the formation of fully mature muscle tissue during in vivo differentiation. These observations are consistent with data on the phenotype of Pax7-deficient mice. These mice are born smaller and have significantly reduced muscle mass compared to control mice [33]. Most of them does not develop properly and dies 2 weeks after birth [35]. Additionally, there are no studies analyzing the “maturity” of muscle tissue Pax7−/− mice. Analysis of myogenesis in teratomas allowed us to document the delayed development of muscle tissue due to lack of Pax7.

During embryonic myogenesis, myoblasts fuse and form primary myofibers which are necessary to establish the basic skeletal muscle pattern [40]. Next, fetal myoblasts fuse with each other to form secondary fibers that are initially smaller and surround primary fibers, or fuse with primary fibers [40]. Pax7 was suggested to participate in switching the myoblast differentiation mode from embryonic to fetal program. However, the data documenting Pax7 action as a molecular switch was limited but showing that Pax7 indirectly activates expression of fetal-specific genes [45].

Our previous study documented that similarly as in vitro [4] also in vivo Pax7−/− cells proliferate more vigorously [8], what may explain higher mass of Pax7−/− teratomas. Analysis of the levels of mRNAs encoding factors crucial for generation of mesodermal and myogenic cells, such as *T* encoding Brachyury—factor synthesized by primitive streak, i.e., mesoderm cells, *Pax3*, *Nfatc4*—embryonic myoblasts, and *Nfix*, *Eno3*, *Mck*, *Mef2a*, *Itga7*—fetal myoblasts markers, uncovered further differences. In Pax7−/− teratomas, the level of *T* mRNA was higher. Also, Pax3 expression was upregulated. A higher level of *PAX3* expression was also observed in the muscles of patients carrying loss of function mutation in *PAX7* gene, leading to a myopathy of variable severity [66]. On the other hand, the levels of mRNAs coding the fetal myoblast markers were lower in Pax7−/− teratomas. Previous study documented that Pax7 activates transcription of *Nfix* mRNA and thus initiates the fetal program in in vitro cultured embryonic myoblasts [45]. Disturbances of *Nfix* expression led to the disruption of fetal myoblast differentiation and their fusion by decreasing the expression of *MyoG* and *Itga7* [40]. In consequence, less myoblasts was able to fuse and resulting myotubes were considerably smaller. Possibly, other factors besides Pax7 may activate *Nfix* during fetal and perinatal development, but this issue was not investigated by us.

Our results document that in vitro Pax7−/− ESCs differentiate to embryonic myoblasts more readily than wild type ones. It is possible that in the absence of functional Pax7 initiation of myogenic differentiation is somehow facilitated and for this reason the expression of mesoderm embryonic myoblast markers is higher. In vivo, at the advanced stages, myogenic differentiation “slows down” in Pax7−/− ESCs what resulted in the decreased expression of markers specific for fetal-type cells and in the smaller area occupied by cells/fibers expressing skeletal myosin. Such phenotype may be associated with a lower ability of differentiating cells to form mature myofibers. Thus, we propose that Pax7 mediates the molecular switch from embryonic to fetal program of myoblast differentiation. Nevertheless, this conclusion needs to be validated by more precise methods. It should also be taken into account that teratomas are built of stochastically arranged tissues at different stages of maturity. Most likely, analysis of the transcripts of individual myoblasts, or other cell populations present in teratomas, would give more conclusive gene expression results than whole teratoma analyses. However, so far, results of the analyses of single cells isolated from teratomas were published only ones, which proves the difficulties in applying this method [24].

Pax7−/− mice were described to lack satellite cells and to be characterized by significantly impaired muscle regeneration [67]. Some studies have shown, however, that in skeletal muscles of Pax7−/− mice satellite cells are not completely absent; nevertheless, their muscle regeneration is certainly impaired [36]. Muscles of these mice were also characterized by smaller fibers containing less nuclei, as compared to control mice. To gain the insight into the role of Pax7 in satellite cell formation, we explored expression of genes and proteins specific for these cells, i.e., *Myf5* and *Cdh15* coding M-cadherin. The levels of *Myf5* and *Cdh15* mRNA and proteins were lower in Pax7−/− teratomas, what indicates abnormal formation or maintenance of satellite cell population. In Pax7−/− teratoma muscles, significantly less satellite cells were detected, as compared to control tissue. Surprisingly, in the absence of functional Pax7, the satellite cell niche, i.e., basal lamina organization, was impaired. Possibly, it was a consequence of a lower expression of *Lama1* gene. Loss of laminin alpha 1 was previously reported as a cause of the impairment of satellite cell proliferation and self-renewal [68]. Thus, a significantly lower number of satellite cells might result from inappropriate niche formation. There are 16 laminin isoforms responsible for maintaining proper basement membrane architecture, regulating differentiation, migration, and adhesion properties of muscle cells [69, 70]. The laminins are heterodimers of alpha, beta, and gamma subunit. Interestingly, within the NMJs, the postsynaptic membrane is rich in laminin chains alpha 2, alpha 4, alpha 5, beta 2, and gamma 1 [60–63]. Interactions between the abovementioned laminins are necessary for proper AChR anchorage and NMJ maturation during embryonic development (summarized in [71]). Tamaki et al. reported that different myogenic capacity of in vitro cultured myogenic cells depends on the ability to produce basal lamina proteins [72]. The key factor responsible for basal lamina protein (e.g., laminin alpha 2) synthesis in myogenic cells was not identified, yet. However, Tamaki suggested that Pax7 could be the one [72]. C2C12 myoblasts in vitro cultured on laminin (containing alpha 5, beta 2, and gamma 1 chains) formed myotubes with “maturing” AChR clusters [73]. Laminins may therefore be the extracellular NMJ “organizers” [73]. Moreover, laminin beta 2 interacts with calcium channels and influences the correct distribution of synaptic vesicles in axon endings [74–76]. Laminin-deficient mice exhibit abnormal development of the NMJ presynaptic membrane [75, 76] and abnormal maturation of calcium canals [77]. Laminin alpha 4-defficient mice showed a loss of precise contact between the pre- and postsynaptic part of NMJ [78]. Moreover, *Lama5*−/− mice die between 14 and 17 d.p.c. due to many developmental defects, including cerebral hernia [79]. Patients with congenital myasthenic syndrome, characterized by muscle weakness and NMJ conduction disorders, were found to have a homozygotic mutation in the *Lama5* and *Lama1* gene, encoding laminin alpha 1 [80]. Abnormalities in the structure of AChR were not observed, and these receptors were present in the motoneuron endings in the presynaptic part of NMJ [80]. Laminins probably promote maturation of the postsynaptic part by autocrine action on the transmembrane dystroglycan receptor [64]. Moreover, laminins alpha 1 and alpha 5 interact with motoneuron endings what stimulates NMJ organization and proper maturation of myotubes to myofibers [64]. The key role of matrix proteins in the development of skeletal muscles is emphasized by the fact that in humans a deficit of basement membrane proteins (including laminins) may lead to the development of incurable muscle dystrophies. Muscles of Pax7-null mice are characterized by smaller fibers containing less nuclei [36]. In our study, muscles of Pax7−/− teratomas presented very similar phenotype. So far, there was no data showing the direct effect of the lack of functional Pax7 on NMJ organization. Our whole volume analysis (samples prepared with iDISCO) showed impaired NMJ system in Pax7−/− muscles. Thus, the finding that myofibers (both NMJ^pos^ and NMJ^neg^) in Pax7−/− teratomas are characterized by smaller dimension than those generated in control teratomas is a novel observation. Also, in Pax7−/− teratomas, myofibers of small diameter (< 20 μm) were NMJ^pos^. However, the proportion of larger, “more mature” NMJ^pos^ myofibers (> 20 μm) was significantly decreased in Pax7−/− teratomas. Moreover, in Pax7−/− teratomas, large muscle fibers (> 30 μm) were mostly NMJ^neg^. It should be also considered that impaired myogenesis in Pax7−/− teratomas could be a result of AChR loss in the cell membrane of large muscle fibers and defective NMJ formation. AChR loss has been previously described as a cause of neuromuscular disease called myasthenia gravis (reviewed in [81]). Summarizing, we hypothesize that the lack of functional Pax7 may affect NMJs through aberrant basal lamina composition.

Molecular analysis of neuron-specific factors, such as *RbFox3* and *Otx2*, revealed lower level of their expression in Pax7−/− teratomas. Importantly, many lines of evidence document that in developing embryo formation of neural tissue is also regulated by Pax7 [29] and Pax7-deficient mice are characterized by cephalic neural crest cell defects [33]. It is also possible that decreased number of satellite cells in muscle tissue of Pax7−/− teratomas impacts the innervation. In 2015, Liu and coworkers speculated that satellite cells may contribute to the regeneration of the NMJs, in response to denervation [82], and that satellite cell deficiency accelerated the NMJ degeneration associated with sarcopenia in mice [83]. In 2018, Bachman et al. showed that prepubertal loss of satellite cells impaired myofiber growth, and skeletal muscle function, but was not associated with disruption in NMJ integrity [65]. However, NMJ defects may require a longer period of time to be manifested, i.e., satellite cell depletion at earlier stages may target the addition of myonuclei to form myofibers and lead to NMJ disruption.

Although formation of primary (embryonic) myotubes is nerve independent [84–86], neuromuscular transmission and nerve-derived signals play important role in myofiber maturation, maintenance, and functioning at the later stages of development [87, 88]. Denervation of the muscle during secondary myogenesis results in a dramatic and permanent deficit in the number of secondary myotubes resulting in muscle size reduction and the dependence of secondary myogenesis on the innervation of myofibers [85–87].

## Conclusions

Our results show that in vivo Pax7−/− ESCs differentiate to embryonic myoblasts more readily than Pax7+/+ cells. Lack of functional Pax7 leads to a disturbance in the formation of fetal myoblasts, what then results in less effective myotube formation, myofiber maturation, and formation of satellite cell population. It seems that in the absence of functional Pax7 initiation of myogenic differentiation is facilitated and, as a result, the expression of mesoderm embryonic myoblast markers is upregulated. Thus, we postulate that Pax7 mediates molecular switch between embryonic to fetal program of myoblast differentiation. Moreover, the lack of functional Pax7 is associated with aberrant formation of neuromuscular junctions what results of lower differentiation potential of Pax7−/− ESCs at advanced stages of myogenesis. Although Pax7 is not essential for in vivo myogenic differentiation of pluripotent cells, the lack of its expression impairs advanced stages of myogenesis. Moreover, lower potential of in vivo ESC myogenic differentiation is caused by abnormal formation of myofiber basement membrane and defective NMJ organization due to lack of Pax7.

## Supplementary information

**Additional file 1: Movie 1.** Whole volume analysis of NMJs in Pax7+/+ teratomas. 3D visualization movie, BTX – yellow.

**Additional file 2: Movie 2.** Whole volume analysis of NMJs in Pax7−/− teratomas. 3D visualization movie, BTX – yellow.

## Data Availability

All data generated or analyzed during this study are included in this published article and its supplementary information files.

## Abbreviations

AChE: Acetylcholine esterase
AChR: Acetylcholine receptor
BTX: α-Bungarotoxin
Mck: Muscle creatine kinase
EBs: Embryoid bodies
Eno3: Enolase 3
ESCs: Embryonic stem cells
iPSCs: Induced pluripotent stem cells
Itga7: Integrin subunit alpha 7
Mef2a: Myocyte enhancer factor 2A
Mrf4: Muscle-specific regulatory factor 4
MRFs: Myogenic regulatory factors
Myf5: Myogenic factor 5
Myh: Myosin heavy chains
MyoD: Myogenic differentiation 1
MyoG: Myogenin
Nfatc4: Nuclear factor of activated T cells
Nfix: Nuclear factor one X
NMJ: Neuromuscular junction
PSCs: Pluripotent stem cells
skMyh: Skeletal muscle myosin heavy chains
